# Supplementary material for: Electrophysiological Properties from Computations at a Single Voltage: Testing Theory with Stochastic Simulations
Source: Entropy (Basel). 2021 May 6;23(5):571. doi: 10.3390/e23050571 (PMC8148522; doi:10.3390/e23050571)
Supplement: Supplementary file 1 [file entropy-23-00571-s001.zip › entropy-1041047-supplementary.pdf]

Supplementary Materials: Supplemental Material for Electrophysiological Properties from Computations at a Single Voltage: Testing Theory with Stochastic Simulations

Michael A. Wilson [1,2] and Andrew Pohorille [1,3,*]

S1. Derivation of the PMF by way of the Committor Probability Method

The integrated form of the ED equation for the forward current takes the form

$$J^f = \frac{\rho^f(z_{min})e^{\beta E(z_{min})} - \rho^f(z)e^{\beta E(z)}}{\int_{z_{min}}^{z} \frac{\exp[\beta E(z')]}{D(z')} dz'}. \tag{S1}$$

Consider a hypothetical channel that extends only from z_{min} to $z < z_{max}$. This is a first passage problem—an ion is adsorbed at the x,y-plane that is located at z and its further fate is not considered. The forward current through this truncated channel, which we abbreviate J_z^f, is different from J^f, even though it still corresponds to a steady state. Analogously to Eq. S1, J_z^f is equal to

$$J_z^f = \frac{\rho_z^f(z_{min})e^{\beta E(z_{min})} - \rho_z^f(z)e^{\beta E(z)}}{\int_{z_{min}}^{z} \frac{\exp[\beta E(z')]}{D(z')} dz'}. \tag{S2}$$

Here, we take advantage of the fact that the PMF, the applied voltage and diffusivity is the same for both systems.

If $J^f > 0$ we take the ratio of J^f to J_z^f in Eq. S1 and Eq. S2. Note the difference from CWDM, where the ratio of forward and backward currents is taken. We obtain

$$\frac{J^f}{J_z^f} = \frac{\rho^f(z_{min})e^{\beta E(z_{min})} - \rho^f(z)e^{\beta E(z)}}{\rho_z^f(z_{min})e^{\beta E(z_{min})} - \rho_z^f(z)e^{\beta E(z)}}. \tag{S3}$$

This equation can be rewritten as

$$\frac{J^f}{J_z^f} = \frac{\rho^f(z_{min}) - \rho^f(z)e^{\beta \Delta E(z,z_{min})}}{\rho_z^f(z_{min}) - \rho_z^f(z)e^{\beta \Delta E(z,z_{min})}}, \tag{S4}$$

where

$$\Delta E(z, z_{min}) = E(z) - E(z_{min}).$$

All ions that reach z contribute to J_z^f. Among them, a fraction will reach z_{max} contributing to J^f. In other words, ions that reach z have a probability $P(z)$ of continuing to z_{max} and contributing to J^f. This probability is precisely the forward referenced committor probability. This means that

$$J^f = P(z)J_z^f. \tag{S5}$$

When this relation is substituted to Eq. S4 we obtain

$$P(z) = \frac{\rho^f(z_{min}) - \rho^f(z)e^{\beta \Delta E(z,z_{min})}}{\rho_z^f(z_{min}) - \rho_z^f(z)e^{\beta \Delta E(z,z_{min})}}. \tag{S6}$$

or, after rearrangements

$$\exp[\beta\Delta E(z, z_{min})] = \frac{\rho^f(z_{min}) - P(z)\rho^f_z(z_{min})}{\rho^f(z) - P(z)\rho^f_z(z)}. \tag{S7}$$

In analogy to Eq. S5

$$P(z)\rho^f_z(z) = \rho^f(z_{max}), \tag{S8}$$

which yields

$$\exp[\beta\Delta E(z, z_{min})] = \frac{\rho^f(z_{min}) - P(z)\rho^f_z(z_{min})}{\rho^f(z) - \rho^f(z_{max})}. \tag{S9}$$

All ions that enter the channel at z_{min} either return to z_{min} or exit at z_{max}. For the truncated channel, the same ions either return to z_{min} or are absorbed at z. If the densities are binned as histogram then at the limit of small bins

$$\rho^f(z_{min}) + \rho^f(z_{max}) = \rho^f_z(z_{min}) + \rho^f_z(z) = \rho^f_z(z_{min}) + \frac{\rho^f(z_{max})}{P(z)}. \tag{S10}$$

This provides an equation for $\rho^f_z(z_{min})$ that can be substituted to Eq. S7. This yields

$$\exp[\beta\Delta E(z, z_{min})] = \frac{\left[\rho^f(z_{min}) + \rho^f(z_{max})\right][1 - P(z)]}{\rho^f(z) - \rho^f(z_{max})}. \tag{S11}$$

Since $\rho^f(z_{min}) >> \rho^f(z_{max})$, this equation simplifies

$$\exp[\beta\Delta E(z, z_{min})] = \frac{\rho^f(z_{min})\,[1 - P(z)]}{\rho^f(z) - \rho^f(z_{max})}. \tag{S12}$$

This is precisely Eq. 13 for reconstructing the PMF from forward simulations.

In the backward direction, this equation takes the form

$$\exp[\beta\Delta E(z, z_{max})] = \frac{\rho^b(z_{max})\left[1 - P^b(z)\right]}{\rho^b(z) - \rho^b(z_{min})}, \tag{S13}$$

where the forward and backward committor probabilities, $P^f(z) = P(z)$ and $P^b(z)$, respectively, are related

$$P^b(z) = 1 - P^f(z) = 1 - P(z). \tag{S14}$$

Since

$$\Delta E(z, z_{max}) = \Delta E(z, z_{min}) - \Delta E(z_{max}, z_{min}), \tag{S15}$$

Eq. S13 becomes

$$\exp[\beta\Delta E(z, z_{min})] = \exp[\beta\Delta E(z_{max}, z_{min})]\frac{\rho^b(z_{max})P(z)}{\rho^b(z) - \rho^b(z_{min})}, \tag{S16}$$

which is Eq. 14.

S2. Detailed derivation of the equation for I-V dependence

From Eqs. 16 and 17, the ratio J_μ / J_ν is

$$\frac{J_\mu}{J_\nu} = \frac{\rho_\mu(z_{min}) - \rho_\mu(z_{max})\exp\left[\beta(E_\nu(z_{max}, z_{min})\right] + \beta q(\mathcal{E}^{el}_\nu - \mathcal{E}^{el}_\mu)\int_{z_{min}}^{z_{max}} \rho_\mu(z)\exp\left[\beta\Delta E_\nu(z, z_{min})\right] dz}{\rho_\nu(z_{min}) - \rho_\nu(z_{max})\exp\left[\beta\Delta E_\nu(z_{max}, z_{min})\right]}, \tag{S17}$$

where $\Delta E_\nu(z_2, z_1)$ is defined according to Eq. 11 and $V_\mu(z)$ and $V_\nu(z)$ according to Eq. 3. This equation can be rewritten as:

$$J_\nu\left[\rho_\mu(z_{min}) - \rho_\mu(z_{max})\exp\left[\beta\Delta E_\nu(z_{max}, z_{min})\right] + \beta q(\mathcal{E}^{el}_\nu - \mathcal{E}^{el}_\mu)\int_{z_{min}}^{z_{max}} \rho_\mu(z)\exp\left[\beta\Delta E_\nu(z, z_{min})\right] dz\right] = J_\mu\left[\rho_\nu(z_{min}) - \rho_\nu(z_{max})\exp\left[\beta\Delta E_\nu(z_{max}, z_{min})\right]\right]. \tag{S18}$$

In the limit of small bin size, $\rho_\mu(z_{max})$ and $\rho_\nu(z_{max})$ measure the number of ions leaving the channel at z_{max}, i.e. measure the currents.

$$\rho_\mu(z_{max}) = \alpha J_\mu, \tag{S19}$$

$$\rho_\nu(z_{max}) = \alpha J_\nu. \tag{S20}$$

In the last two equations, α is a constant in length/time to ensure the correct conversion between the boundary densities and currents.

Let's abbreviate the number density of ions entering the channel into the first bin as $\rho_{in}(z_{min})$. Then, taking advantage of Eqs. S19 and S20,

$$\rho_\mu(z_{min}) = 2\rho_{in}(z_{min}) - \alpha J_\mu \tag{S21}$$

and

$$\rho_\nu(z_{min}) = 2\rho_{in}(z_{min}) - \alpha J_\nu. \tag{S22}$$

The last two equations reflect the fact that in the steady state all ions that enter the channel through the z_{min} plane also exit through the same plane with the exception of those that successfully cross the channel.

After substituting Eqs. S19 - S22 to Eq. S18, we obtain

$$\begin{aligned} 2J_\nu\rho_{in}(z_{min}) - \alpha J_\nu J_\mu - \alpha J_\nu J_\mu \exp\left[\beta\Delta E_\nu(z_{max}, z_{min})\right] \\ + J_\nu\beta q(\mathcal{E}^{el}_\nu - \mathcal{E}^{el}_\mu)\int_{z_{min}}^{z_{max}} \rho_\mu(z)\exp\left[\beta\Delta E_\nu(z, z_{min})\right] dz = \\ 2J_\mu\rho_{in}(z_{min}) - \alpha J_\mu J_\nu - \alpha J_\mu J_\nu \exp\left[\beta\Delta E_\nu(z_{max}, z_{min})\right], \end{aligned} \tag{S23}$$

which simplifies to

$$J_\nu\left[2\rho_{in}(z_{min}) + \beta q(\mathcal{E}^{el}_\nu - \mathcal{E}^{el}_\mu)\int_{z_{min}}^{z_{max}} \rho_\mu(z)\exp\left[\beta\Delta E_\nu(z, z_{min})\right] dz\right] = 2J_\mu\rho_{in}(z_{min}). \tag{S24}$$

We define

$$g_\mu(z) = \rho_\mu(z)\exp\left[\beta\Delta E_\mu(z, z_{min})\right]. \tag{S25}$$

Then

$$J_\nu \left[2\rho_{in}(z_{min}) + \beta q(\mathcal{E}^{el}_\nu - \mathcal{E}^{el}_\mu) \int_{z_{min}}^{z_{max}} g_\mu(z) \exp\left\{ \beta q \left[V_\nu(z) - V_\mu(z) \right] \right\} dz \right] = 2 J_\mu \rho_{in}(z_{min}) \quad \text{(S26)}$$

or

$$\frac{J_\mu}{J_\nu} = 1 + \frac{\beta q(\mathcal{E}^{el}_\nu - \mathcal{E}^{el}_\mu) \int_{z_{min}}^{z_{max}} g_\mu(z) \exp\left\{ \beta q \left[V_\nu(z) - V_\mu(z) \right] \right\} dz}{2\rho_{in}(z_{min})}. \quad \text{(S27)}$$

If we assume that the number of ions entering the channel is much larger that the number of ions crossing the channel, an approximation that has been shown to be accurate in practice, then $2\rho_{in}(z_{min})$ can be replaced by $\rho_\mu(z_{min})$ (see Eq. S21). Then:

$$\frac{J_\mu}{J_\nu} = 1 + \frac{\beta q(\mathcal{E}^{el}_\nu - \mathcal{E}^{el}_\mu) \int_{z_{min}}^{z_{max}} g_\mu(z) \exp\left\{ \beta q \left[V_\nu(z) - V_\mu(z) \right] \right\} dz}{\rho_\mu(z_{min})},$$

which directly leads to Eq. 18 in which

$$f_\mu(z) = \frac{g_\mu(z)}{\rho_\mu(z_{min})}.$$

S3. Calculating committor probability

Among ions that entered the channel through the z_{min}-plane, $N^f(z)$ reached the point z in the channel. Since this is a first-passage problem, each ion is counted only once. In particular, the number of ion that reach z_{max} is $N^f(z_{max})$. Taking advantage of these definitions, the forward committor probability, $P^f(z)$, is expressed as

$$P^f(z) = N^f(z_{max})/N^f(z). \quad \text{(S28)}$$

Analogously, the backward committor proability, $P^b(z)$ can be written as

$$P^b(z) = N^b(z_{min})/N^b(z), \quad \text{(S29)}$$

where $P^f(z)$ and $P^b(z)$ are connected via Eq. S14.

In a stochastic process, there is no memory about the history of an ion that reached z. Therefore, forward and backward committor probabilities can be combined. The total number of ions that reach z is $N^f(z) + N^b(z)$ and the total number of ions that leave through (are absorbed at) the z_{max}-plane is $N^f(z_{max}) + N^b(z) - N^b(z_{min})$. This yields the following formula for the forward referenced committor probability

$$P(z) = \frac{N^f(z_{max}) + N^b(z) - N^b(z_{min})}{N^f(z) + N^b(z)}. \quad \text{(S30)}$$

S4. Free Energy Surfaces

To determine the accuracy of the recently developed approaches to calculating the I-V curves (see Section S2), we utilized PMFs for ions crossing three different channels. Below, we provide details on how these PMFs were obtained. However, as we stressed and explained in the Introduction, no conclusions from this study depend on how accurately the calculated PMFs reproduce the actual PMFs experienced by the ions.

We use a previously reported model of the TTX channel [1,2], an unpublished calculation on the p7 viroporin from the Hepatitis C virus, and an unpublished calculation on the pH-gated Na^+ channel

GLIC. Here, we provide brief details of the simulations that were used to generate the input PMFs. Pictures of the channels are shown in in Fig. S1

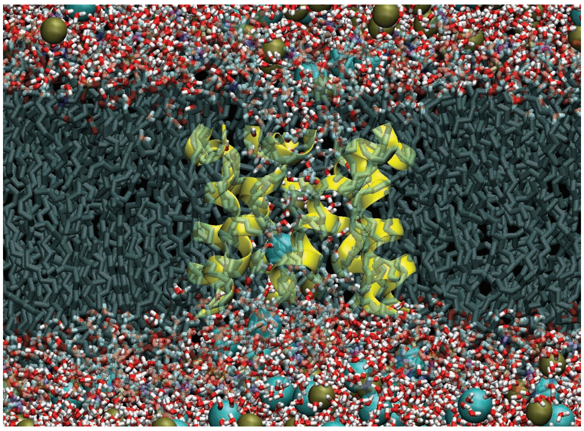

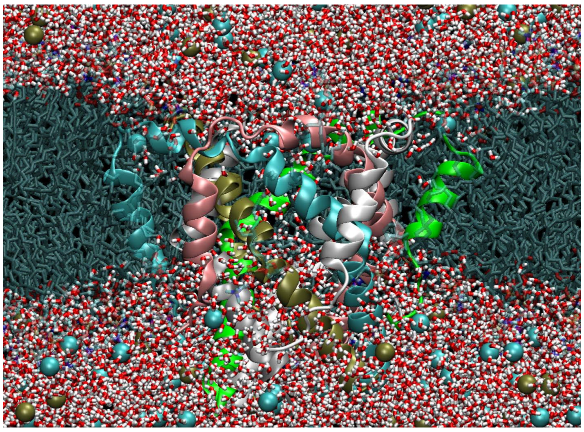

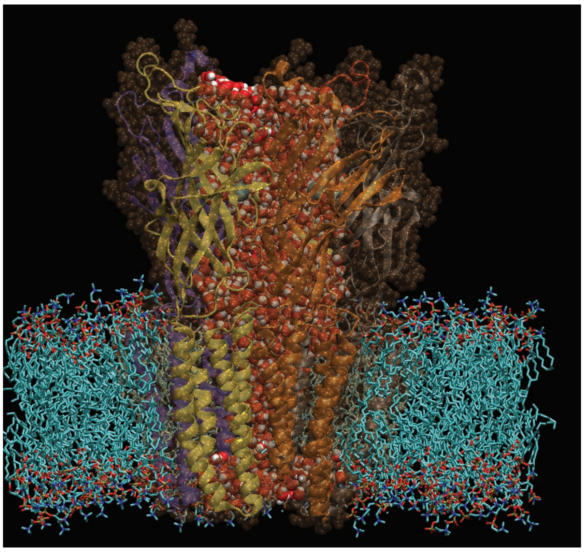

Figure S1. Pictures from MD simulations of (top left) TTX showing a seven α-helix bundle (yellow ribbons) embedded in a POPC membrane (dark blue licorice with gold, red and blue licorice in the head group region), water (rad and white licorice), K^+ (gold) and Cl^- (blue) ions; (top right) p7 showing the 6 subunits (different colored ribbons) embedded in a POPC membrane and water molecules (red and white CPK); and (bottom left) GLIC (monomerics in different color ribbons plus transparent gold CPK showing the spatial extent of the protein) in a POPC membrane. Waters and ions have been removed except for waters in the pore and vestibule of the protein.

The MD simulations of TTX [1,3] were carried out at the experimental concentration of 1 M KCl [4]. Simulations of p7 were carried out at 0.5 M (Shannon, Wilson and Pohorille, unpublished). The GLIC simulations were carried out at the physiological concentration of 140 mM NaCl (Wilson and Pohorille, unpublished). Preliminary calculations on GLIC were also performed at a higher concentration of 500 mM, but increased concentration had little effect on the conductance, likely due to saturation of Na^+ in the vestibular region.

In all three simulations, the protein was embedded in a POPC membrane with water and salt concentrations as noted on both sides of the lipid bilayer. In all simulations, the TIP3P model of water[5] was used. In the TTX simulations, the CHARMM27 [6] and the CHARMM27 with CMAP corrections [7] were used to represent POPC and the protein, respectively. In the p7 and GLIC simulations, the CHARMM36 potentials [8] were used to represent both proteins and lipids. After equilibration, all simulations were carried out in an NVT ensemble with an applied potential, noted below, using Ewald sums and periodic boundary conditions with a time step of 2 fs. In all simulations, backbone restraints were applied to the protein to prevent collapse of the pore.

TTX: (see Wilson, *et al.*[1] for details), The simulation box was: 84.854 Å × 84.2246 Å × 106.322 Å. The system contained 16628 water molecules, 300 ions each of K^+ and Cl^-, 204 POPC molecules, and 7 TTX monomers each containing 18 residues (255 atoms). A model of the TTX channel consisting of 7 straight helices that we developed was used. A MD trajectory 900 ns with applied voltage of 50

mV was generated by way of NAMD. Additional, shorter simulations were carried out at 0 V, -50 mV (500 ns) and 100 mV (315 ns). The currents were calculated at all these voltages. At 50 mV, the numbers of crossing events observed were: K^+: 198 with the field, 32 against the field; Cl^-: 84 with the field, 10 against. The PMF was obtained from nonequilibrium simulations at applied voltage and from separate adaptive biasing force (ABF) calculations [9]. In the latter case, the transmembrane region was subdivided into 7 strata or windows, 6 Å wide each overlapping its neighboring windows by 2 Å. Then, trajectories 60 ns long were obtained with an ion constrained in each window, and the free energy profile was constructed by integrating the average force across the pore region. The PMF from both methods were found to be in good agreement[1]. The PMF from the latter simulations was used in this study.

p7: A 2.06 μsec trajectory for NMR structure (PDB: 2M6X)[10] of p7 at applied voltage 140 mV was obtained on the Anton machine at CMU. Additional shorter trajectories were carried out at voltages of -140 mV, -105mV, -70 mV, -35 mV, 0 mV, 35 mV, 70 mV, and 105 mV (300 ns, each). The simulation box was 112.285 Å $\times$ 113.792 Å $\times$ 103.052 Å. The system contained 27864 water molecules, 265 K^+ and 301 Cl^- ions (to balance the protein charge), 349 POPC molecules, and 6 p7 monomers each containing 63 residues (1000 atoms) with ACE and CT2 terminal blocking groups. The numbers of crossing events at 140 mV were: K^+: 50 with the field, 0 against the field; Cl^-: 625 with field, 5 against the field. The p7 PMF was obtained over the range [-22,19] from ABF using 7 windows of 8 Å width, each overlapping its neighboring windows by 2 Å, with a 250 ns trajectory in each window. The PMF was also calculated from the 2.06 μsec trajectory at 140 mV from the the non-equilibrium CPM and CWDM methods outlined above (Shannon, Wilson, Chipot and Pohorille, unpublished). The results from these three methods were found to be in good agreement.

GLIC: A 7.68 μsec trajectory for X-Ray structure of GLIC (PDB: 4HFI) [11] at applied voltage 100 mV was obtained on the Anton machine at CMU. The simulation box was 121.703 Å $\times$ 121.703 Å $\times$ 171.386 Å. The system contained 60806 water molecules, 147 K^+ and 117 Cl^- ions (to balance the protein charge), 400 POPC molecules, and 5 GLIC monomers each containing 312 residues (5083 atoms) with ACE and CT3 terminal blocking groups. The numbers of crossing events were: Na^+: 23 with field, 1 against field; Cl^-: no crossing events observed. The GLIC free energy profile was determined from non-equilibrium simulations using the CPM approach.

S5. Diffusivity Calculations

As noted in the text, several methods are available to calculate diffusivities. Although the diffusivity drops out of the expressions for free energy and current in the formulations used here, it is needed to link time scales of the stochastic and MD simulations (see Section 7). Once the PMF had been obtained, the diffusivity at a number of points along the channel was calculated. To do so, configurations from the PMF calculation with the ion located within ± 0.5 Å of the selected value of z were used. Then, for each configuration, a set of MD trajectories was initiated in which the force due to the PMF is subtracted from the forces acting on the ion. The resultant ion trajectory was due only to the random forces acting on the ion. This means that diffusivity can be extracted from Einstein's relation. For each point z_i, the quantity $< (z(t) - z(0))^2 >$ was typically calculated from 100 trajectories of 100 ps, each. If the process is diffusive then, at longer times, $< (z(t) - z(0))^2 >$ is linear in t with slope $2D(z_i)$. Thus, calculating diffusivity at each value of z required approximately 10 ns of MD trajectories. Usually, we computed the diffusivities at 3-7 points, depending on the shape of the pore. Results of diffusivity calculations for the channels considered here are listed below.

TTX Calculations of diffusivity [1], led to the conclusion that this quantity was constant along z in the channel (no statistical basis for rejecting this as the null hypothesis). The average values of the diffusion coefficient were D_K = 0.97 Å^2/ps and D_{Cl} = 0.80 Å^2/ps for K^+ and Cl^-, respectively..

p7 Diffusivities were calculated at 4 points along the channel, -12 Å,-5 Å, 2.5 Åand 7 Å(with respect to the protein center-of-mass). Their values were, respectively, 0.07 Å^2/ps, 0.09 Å^2/ps, 0.11 Å^2/ps and

0.09 Å^2/ps, which gives no statistical basis to reject the hypothesis that diffusivity in the channel is constant. In the stochastic simulations we used an average diffusion coefficient of D_{Cl} = 0.09 Å^2/ps.

GLIC Diffusivity was calculated as 0.01 Å^2/ps and 0.008 Å^2/ps at points -1 Å and -9 Å (with respect to the protein COM), respectively . These points correspond to the narrowest points in the channel region and, therefore, provide a good test if diffusion in the channel is Fickean rather than single file. A diffusion coefficient of 0.009 Å^2/ps was used in stochastic simulations.

S6. Connecting fluxes from crossing statistics to electrical displacement currents

We determine the ion fluxes from their crossing statistics. Ions can cross the pore in the direction of the electric field, or against it. An ion is counted as crossing the pore only if it enters on one side of the pore and exits the other side. Many ions enter the pore region and exit the same end. These ions *do not* contribute to the crossing statistics.

An alternative to this approach is to calculate the displacement currents crossing the simulation box [12–14]. Consider the instantaneous current in the simulation cell [12]

$$I(t) = \frac{1}{L_z} \sum_{\text{all atoms}} q_i \dot{z}_i(t),$$

where L_z is the length of the simulation cell in the z-direction, perpendicular to the membrane, q_i is the charge on the ith atom in the system, and $\dot{z}_i(t)$ is the velocity in the z-direction of the ith atom at time t. As the trajectory is stored only every 10–50 ps, we consider the integrated form of this equation:

$$Q(t) = \frac{1}{L_z} \sum_{\text{all atoms}} q_i [z_i(t) - z_i(0)],$$

where z_i is the position of the ith atom, and $Q(t)$ is the cumulative charge. This cumulative charge can be broken down into the cumulative charges for the individual components of the system,

$$Q_\alpha(t) = \frac{1}{L_z} \sum_{i_\alpha} q_{i_\alpha} [z_{i_\alpha}(t) - z_{i_\alpha}(0)],$$

where α runs over the components (α = Na^+ or K^+, Cl^-, water atoms, lipid atoms and protein atoms), q_{i_α} is the charge on atom i in component group α, and $z_{i_\alpha}(t)$ is its position. If the system is in a steady state, then $Q(t)$ should increase linearly with time with the slope equal to the total current in the system. The individual $Q_\alpha(t)$ are also expected to grow linearly with time or be equal to zero.

in Fig. S2, we plot $Q_\alpha(t)$ for TTX and p7. First, we observe that the lipids and proteins do not contribute to the total current. As the center of the system is defined by the protein and lipid, this is expected as the net displacement the charges of those components can experience is small. Therefore, the net displacement charge for these components of the system fluctuates around zero. Second, water molecules are charge neutral and the net translocated charge carried by the water molecules is zero. Thus, the total displacement charge due to water molecules is also small and fluctuates around zero. Third, for the ions, we note that counting channel crossing events to obtain the cumulative charge is almost identical to the results from the integrated current equation. Therefore, the current estimated from the slope of the cumulative count of crossing events is the same as estimated from the integrated current equation, which, in the long-time limits, yields the total electrical current. The results for Na^+ in GLIC (not shown) are similar, although the statistics are markedly worse, as only 22 net Na^+ crossing events were observed. We note that these are fairly simple pores; we do not observe ion binding in the pore region and do not expect non-diffusive behavior. Consequently, the lifetime of ions in the pore is much shorter than the simulation time scale.

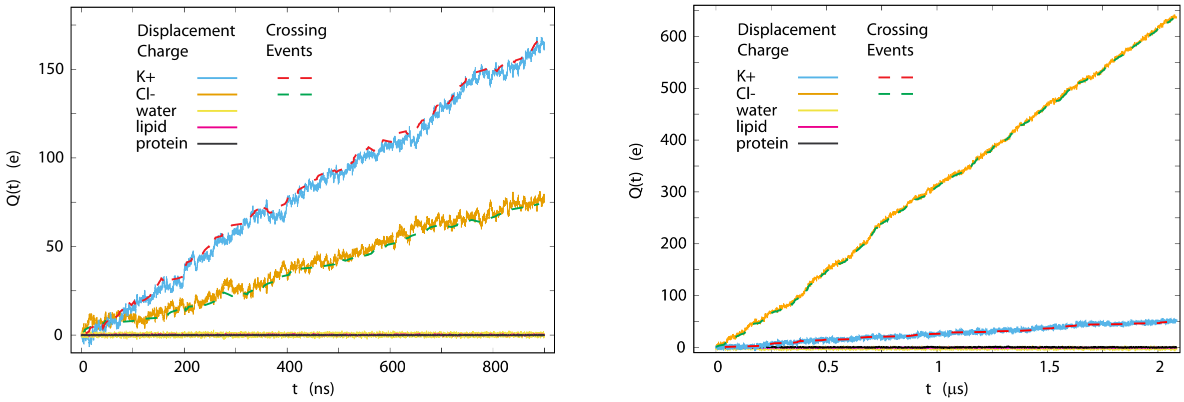

Figure S2. Total displacement charge for TTX (**a**) , and for p7 (b). The integrated current equation for K^+ (blue) and Cl^- (gold) is compared with cumulative charge from crossing events for K^+ (red) and Cl^- (green). The integrated currents for water (yellow), lipid (magenta) and protein (black) are also shown. The larger fluctuations in the integrated currents for TTX compared with p7 are due to the smaller scale in TTX (Q 200 e) vs p7 (Q 650 e) and the finer sampling resolution for TTX (10 ps) vs. p7 (50 ps).

S7. Synthetic Datasets from Stochastic Simulations

We investigate how the ion crossing statistics affect the accuracy of the PMFs and the I-V curves for ions in the channels studied here. To do this, we carry out stochastic simulations of ions traversing the channel in the presence of the PMF to mimic the ion crossing statistics observed in the MD simulations. We consider PMF only in the channel region, and we collect the crossing statistics by initiating trajectories at each end of the channel.

Trajectories were generated using the Brownian dynamics algorithm [15,16]:

$$z(t+h) = z(t) + \frac{D(z)F(z)}{k_B T}h + \frac{dD(z)}{dz} + \sqrt{(2D(z))}dW \tag{S31}$$

where $z(t+h)$ is the position at time $t+h$ given that the ion was at z at time t, $D(z)$ is the diffusivity, $F(z)$ is the force equal to the negative gradient of the free energy surface (PMF plus applied electric field), dW is a Gaussian process with variance 1, T is temperature and k_B is the Boltzmann constant. The additional force term due to the derivative of $D(z)$ is required, as the ED equation is a form of the Smoluchowski equation, which has a fluctuation-dissipation term connecting $F(z)$ and the stochastic force [15,17]. If the diffusivity is position-independent, equal to the diffusion coefficient, $< D >$, the derivative term vanishes and the standard Brownian Dynamics equation is recovered. Since the diffusivity appears to be nearly constant in all three channels considered here, the values of $< D >$ listed in Section 5 were used. If a position-dependent $D(z)$ is used, the density profiles and committor probabilities are somewhat different than those for constant $< D >$. However, the reconstructed free energy surface is the same. The Brownian dynamics approach used here to generate trajectories on a 1-dimensional free energy surface (PMF + potential due to applied field) is distinct from Brownian Dynamics method for calculating ionic currents in channels [18–23].

If the PMF is defined over the range $[z_{min}, z_{max}]$ then we set absorbing boundaries at z_{min} and z_{max}. We initiate trajectories at these two points and call them, respectively, forward and backward trajectories. If we initiate N^f stochastic trajectories at z_{min}, we denote n^f as the number of trajectories that cross the pore region and are absorbed at z_{max}. Similarly, if we initiate N^b trajectories at z_{max}, n^b trajectories will cross the pore region and be absorbed at z_{min}, while N-n^b trajectories will be reabsorbed at z_{max}. n^f and n^b are the number of forward and backward crossing events, respectively. The values of n^f (n^b) will depend on N^f (N^b) and the PMF, as well as other parameters, such as the diffusivity, $D(z)$, and the temperature, T. We choose values of N^f and N^b such that the number of crossing events n^f and n^b approximately correspond to the numbers of crossing events in both directions seen in the MD

simulations. This set of N^f and N^b simulations constitutes one dataset. We then carry out simulations to generate m datasets. For each dataset, we calculate the 1-sided density profiles, $\rho^f(z)$ and $\rho^b(z)$ and the forward and backward committor probabilities, $P^f(z)$ and $P^b(z)$. We then investigate the distribution of outcomes over the m independent datasets to determine the size of the statistical errors.

References

1. Wilson, M.A.; Nguyen, T.H.; Pohorille, A. Combining molecular dynamics and an electrodiffusion model to calculate ion channel conductance. *J. Chem. Phys.* **2014**, *141*, 22D519.
2. Pohorille, A.; Wilson, M.A.; Wei, C. Validity of the electrodiffusion model for calculating conductance of simple ion channels. *The Journal of Physical Chemistry B* **2017**, *121*, 3607–3619.
3. Pohorille, A.; Wilson, M.A.; Wei, C. Validity of the Electrodiffusion Model for Calculating Conductance of Simple Ion Channels. *J Phys Chem B* **2017**, *121*, 3607–3619.
4. Duclohier, H.; Alder, G.M.; Bashford, C.; Brückner, H.; Chugh, J.K.; Wallace, B.A. Conductance studies on trichotoxin_A50E and implications for channel structure. *Biophysical Journal* **2004**, *87*, 1705–1710. doi:10.1529/biophysj.104.040659.
5. Jorgensen, W.L.; Chandrasekhar, J.; Madura, J.D.; Impey, R.W.; Klein, M.L. Comparison of simple potential functions for simulating liquid water. *The Journal of Chemical Physics* **1983**, *79*, 926–935. doi:10.1063/1.445869.
6. Feller, S.E.; MacKerell, A.D. An improved empirical potential energy function for molecular simulations of phospholipids dynamics simulation of a lipid bilayer. *J. Phys. Chem. B* **2000**, *104*, 7510–7515.
7. MacKerell, A.D., J.; Feig, M.; Brooks, C.L., I. Extending the treatment of backbone energetics in protein force fields: Limitations of gas-phase quantum mechanics in reproducing protein conformational distributions in molecular dynamics simulations. *J. Comp. Chem.* **2004**, *25*, 1400–1415.
8. Best, R.B.; Zhu, X.; Shim, J.; Lopes, P.E.M.; Mittal, J.; Feig, M.; MacKerell, A.D. Optimization of the Additive CHARMM All-Atom Protein Force Field Targeting Improved Sampling of the Backbone ϕ, ψ and Side-Chain χ_1 and χ_2 Dihedral Angles. *J. Chem. Theory Comput.* **2012**, *8*, 3257–3273, [http://pubs.acs.org/doi/pdf/10.1021/ct300400x]. doi:10.1021/ct300400x.
9. Darve, E.; Pohorille, A. Calculating free energies using average force. *The Journal of Chemical Physics* **2001**, *115*, 9169–9183. doi:10.1063/1.1410978.
10. OuYang, B.; Xie, S.; Berardi, M.J.; Zhao, X.; Dev, J.; Yu, W.; Sun, B.; Chou, J.J. Unusual architecture of the p7 channel from hepatitis C virus. *Nature* **2013**, *498*, 521–525.
11. Sauguet, L.; Poitevin, F.; Murail, S.; Van Renterghem, C.; Moraga-Cid, G.; Malherbe, L.; Thompson, A.W.; Koehl, P.; Corringer, P.J.; Baaden, M.; others. Structural basis for ion permeation mechanism in pentameric ligand-gated ion channels. *The EMBO journal* **2013**, *32*, 728–741.
12. Roux, B. The membrane potential and its representation by a constant electric field in computer simulations. *Biophysical journal* **2008**, *95*, 4205–4216.
13. Gumbart, J.; Khalili-Araghi, F.; Sotomayor, M.; Roux, B. Constant electric field simulations of the membrane potential illustrated with simple systems. *Biochim Biophys Acta* **2012**, *1818*, 294–302.
14. Aksimentiev, A.; Schulten, K. Imaging α-hemolysin with molecular dynamics: ionic conductance, osmotic permeability, and the electrostatic potential map. *Biophysical journal* **2005**, *88*, 3745–3761.
15. Pieprzyk, S.; Heyes, D.M.; Brańka, A.C. Spatially dependent diffusion coefficient as a model for pH sensitive microgel particles in microchannels. *Biomicrofluidics* **2016**, *10*, 054118, [https://doi.org/10.1063/1.4964935]. doi:10.1063/1.4964935.
16. Huber, G.A.; McCammon, J.A. Brownian Dynamics Simulations of Biological Molecules. *Trends Chem* **2019**, *1*, 727–738.
17. Risken, H. *The Fokker-Planck Equation*; Springer-Verlag: New York, 1996.
18. Chung, S.H.; Allen, T.W.; Kuyucak, S. Modeling diverse range of potassium channels with Brownian dynamics. *Biophys. J.* **2002**, *83*, 263–277.
19. Noskov, S.Y.; Im, W.; Roux, B. Ion permeation through the α-hemolysin channel: Theoretical studies based on Brownian dynamics and Poisson-Nernst-Plank electrodiffusion theory. *Biophys. J.* **2004**, *87*, 2299–2309.

20. Cheng, M.H.; Coalson, R.D. An accurate and efficient empirical approach for calculating the dielectric self-energy and ion-ion pair potential in continuum models of biological ion channels. *J. Phys. Chem. B* **2005**, *109*, 488–498.
21. Coalson, R.D.; Kurnikova, M.G. Poisson-Nernst-Planck theory approach to the calculation of current through biological ion channels. *IEEE transactions on nanobioscience* **2005**, *4*, 81–93.
22. Maffeo, C.; Bhattacharya, S.; Yoo, J.; Wells, D.; Aksimentiev, A. Modeling and simulation of ion channels. *Chemical reviews* **2012**, *112*, 6250–6284.
23. Flood, E.; Boiteux, C.; Lev, B.; Vorobyov, I.; Allen, T.W. Atomistic Simulations of Membrane Ion Channel Conduction, Gating, and Modulation. *Chem Rev* **2019**, *119*, 7737–7832.

Publisher's Note: MDPI stays neutral with regard to jurisdictional claims in published maps and institutional affiliations.
